# Scabies as a part of the World Health Organization roadmap for neglected tropical diseases 2021–2030: what we know and what we need to do for global control

**DOI:** 10.1186/s41182-021-00348-6

**Published:** 2021-08-16

**Authors:** Amal A. El-Moamly

**Affiliations:** Department of Medical Parasitology, Faculty of Medicine, Suez Canal University, Round Road, Ismailia, Postal Code 41522 Egypt

**Keywords:** Scabies, *Sarcoptes scabiei*, Control, Neglected tropical diseases, Control, Epidemiology, WHO roadmap, Diagnosis, Treatment, Vaccine, Mass drug administration

## Abstract

**Background:**

Scabies is an under-recognized global health problem with an unacceptably high prevalence in many settings worldwide. Fortunately, the World Health Organization (WHO) has formally designated scabies as a neglected tropical disease in 2017, in the hope of increasing awareness and encouraging efforts to eradicate it. Also, scabies has recently been included as part of the WHO roadmap for neglected tropical diseases 2021–2030, aimed at ending the neglect to attain the Sustainable Development Goals.

**Main abstract body:**

This review article places scabies in focus. The literature was reviewed to explore discussions on controversial issues in scabies control, with the aim of clarifying whether global control of scabies is a feasible and worthwhile objective. The existing status of scabies and its burden are discussed along with future prospects for its global control. The article investigates the feasibility of scabies control and provides updates on the various impediments to this goal, such as challenges related to transmission, diagnosis, treatment, and vaccine development. Also examined are relevant research needs, success factors, and reasons for failure. This article aims to increase the global awareness of scabies and promote discussion, enhance coordinated international efforts, and ultimately, enact change at the national and worldwide levels toward the control of this preventable disease of the poor.

**Conclusion:**

Despite the current challenges, scabies control is now within reach. With sustained interventions, continuous resources, and sincere commitment and support, scabies global control appears to be a worthwhile, realistic goal that is potentially achievable in the not so distant future.

## Background

The World Health Organization (WHO) recently acknowledged scabies as one of the neglected tropical diseases (NTDs) [1]. At their meeting in March 2017, the Strategic and Technical Advisory Group (STAG) of WHO for NTDs recommended including scabies in their NTD portfolio [1]. The STAG issued this recommendation based on the recently developed WHO criteria for the inclusion of additional NTDs. The criteria specify that proposed diseases must “i) disproportionately affect populations living in poverty and cause important morbidity and mortality; ii) primarily affect populations living in tropical and sub-tropical areas; iii) be immediately amenable to control, elimination or eradication, and iv) be relatively neglected by research” [1]. The STAG decided that all four criteria were met for scabies. After that, in 2020, and as a step toward further efforts to deal with the neglect to attain the Sustainable Development Goals (SDGs), scabies along with other ectoparasite-causing diseases were included in the updated WHO roadmap for neglected tropical diseases 2021–2030 [2]. Targets, milestones, specific indicators, and critical actions are proposed here to support policies and actions aimed at ending the neglect of these ancient diseases of poverty.

Scabies may be the first reported human disease with a well-known etiology [3, 4]. Its prevalence still shows unacceptably high figures in many settings of the world. It is estimated to affect more than 130 million people globally at any given time [5]. Scabies infestation significantly impacts patients’ quality of life with adverse sequelae, especially in underprivileged and low-resource settings. The parasite incriminated in this highly contagious parasitic skin disease is the burrowing mite *Sarcoptes scabiei* var. *hominis*. It is particularly prevalent among children and older and immunocompromised people, and may lead to considerable morbidity and even mortality in vulnerable individuals especially in resource-poor communities with less access to adequate healthcare [6, 7].

Adding scabies to the global health agenda as an NTD would promote disease awareness and global discussions and collaboration, and encourage much-needed research into various aspects of this disease [8]. In addition to WHO, other world organizations such as the International Alliance for the Control of Scabies (IACS) [9] along with informal groups of concerned researchers such as the Sarcoptes-World Molecular Network [10] are committed to working together to achieve the global control of scabies. However, the recognition of scabies as an NTD has also triggered discussion at the highest levels in the scientific community. These discussions focus on whether the global control of scabies should be our goal at the moment, and on the important challenges and needs in the current and future phases of the fight against scabies [11].

This article places scabies control in focus. It *highlights the global burden of the disease, examines the present situation and future prospects regarding coordinated action to control scabies at a global level, and discusses future prospects.* These plans include improvements in diagnostic methods, therapeutic options, control protocols, and other operational tools and approaches. In addition, this review addresses important research updates and proposes further research needs to support improved control efforts. A literature review was used as the basis for a discussion of controversial matters and challenges in scabies control, with the aim of clarifying whether scabies global control is a feasible and worthwhile objective. This article aims to increase the global awareness of scabies and promote discussion, enhance international efforts, and ultimately, enact change at the national and worldwide levels toward the control of this preventable disease of the poor.

## Why does scabies need focus as a neglected tropical disease?

Scabies can be considered an impediment to the essential human right to live a long life in full health, particularly for poor patients in underprivileged countries [7]. Like other NTDs, scabies can be seen as a sign of poverty and disadvantage; moreover, it causes stigma and discrimination, has a substantial impact on morbidity, and is widely neglected by health providers and researchers. Scabies is considered a public health challenge for underprivileged communities in many low-income and middle-income countries, and also in some resource-rich countries. However, the epidemiological and clinical features of the disease in resource-poor regions are different from high-income settings. In the former, scabies is endemic, but it usually occurs in epidemics in high-income societies [12]. Morbidity is more severe in resource-poor settings and may include life-threatening sequelae. Good control measures in resource-poor regions should prioritize mass treatment at the community level using easy-to-apply drugs, rather than treating individual patients [12].

### Scabies has a high disease burden among the poor

Scabies predominates as one of the commonest skin diseases in underprivileged regions. Despite disagreement on the exact number of affected people, it has been estimated that more than 130 million persons suffer from scabies worldwide at any specified time [5, 13]. Although scabies affects all age groups throughout the world, children and older people in low-resource regions are the most vulnerable groups, among whom the prevalence, morbidity, and secondary complications are greatest [14, 15]. The highest prevalence is reported in locations with hot and humid climates in tropical countries, where the disease is endemic. In different conditions of endemicity, transmission may require prolonged skin contact (lower endemicity) or overcrowded environments (highly endemic settings). Hence, scabies is not viewed as a disease of poor hygiene, but rather as an indirect reflection of poverty and overcrowding, as in prisons, military camps, and boarding schools [9, 12, 16, 17]. Available research has shown that the prevalence of endemic scabies varies significantly between communities and settings, and may reach 20% or more in the overall population [12, 18]. A study from the 1980s in Bangladesh reported an extremely high incidence in preschool children; the great majority of children had experienced at least one infestation per year [19]. In contrast, the disease occurs sporadically in high-income countries, with reported outbreaks in various settings such as nursing homes, child daycare centers, jails, psychiatric facilities, and institutions for older people, where outbreaks can often be tracked back to a single or a few index patients [17, 20–23]. Scabies also occurs in HIV-positive patients, those with “type-1 human T-cell leukemia virus,” and patients with malnutrition [24–26].

The disease burden is prominent especially in developing regions due not only to the infection per se, but also to its complications, which generate extra costs to the healthcare systems in these locations. It was estimated that the direct expense of skin disease exceeds 1.5.million “years lived with disability (YLDS),” while the indirect cost of kidney and heart complications are far higher [9].

The Global Burden of Disease (GBD) 2015 study assessed the extent of scabies in 30 countries [27]. Scabies was responsible for 0.21% of disability-adjusted life-years (DALYs) from 315 included conditions. The major burden was in tropical zones in East, South, and Southeast Asia; Oceania; and Latin America. Of 246 conditions, scabies ranked 101th in terms of age-standardized global DALYs, just after medical error (ranked 98th) and before atrial fibrillation (ranked 102nd) [7, 27].

### Scabies considerably affects quality of life

The effect of scabies on patients’ quality of life goes beyond the hallmark of pruritus [28]. Itching may cause severe sleep disturbance, and the exposed excoriated skin may make social stigma an important concern [29]. According to a study in China, the aspect of patients’ quality of life that was most clearly affected was personal relationships, with the “symptoms and feelings” domain ranking second [30]. Another study from urban slums in Brazil highlighted the significance of the socioemotional characteristics of the disease and their effect on the patients’ quality of life. The patients affected most by socioemotional aspects of scabies were females, 30% of whom reported social exclusion, and the majority reporting feelings of shame [31]. Severe restrictions, sense of shame, impact on free-time activities, and painful stigmatization were very common [31]. Hence, the social influence of scabies should not be neglected, even if the disease has been accepted as a normal part of everyday life in poor patients. It is worth recognizing that the acceptance of infestation as a routine event may in turn affect compliance with treatment in poor settings where the prevalence is high [32].

### Scabies causes serious complications

In addition to its direct impact on quality of patients’ life, scabies is associated with a number of serious complications. Excoriations as a result of severe pruritus predispose sufferers to bacterial skin infections, particularly with *Staphylococcus aureus* and group A *Streptococcus* (GAS) [33, 34]. Pyoderma can progress to potentially fatal invasive bacterial infections such as lymphangitis, lymphadenitis, cellulitis, and sepsis [34]. Long-term consequences such as post-streptococcal glomerulonephritis (PSGN), chronic kidney disease, and possibly rheumatic fever were also noted [35–37]. Repeated episodes of PSGN in childhood are suspected to contribute to chronic severe renal disease later in life [36, 37]. Evidence of kidney damage was found in almost 10% of young patients with infected scabies in poor settings [9]. A link was also found between scabies, PSGN, and chronic kidney disease in patients in remote Australian aboriginal societies [38]. In general, impetigo due to scabies was found to be the main cause of PSGN, hematuria, acute rheumatic fever, and heart diseases among children in low-resource settings [5, 38]. Hence, effective control strategies for scabies may also reduce the incidence of PSGN, renal diseases, and rheumatic heart disease.

### Scabies has considerable financial consequences

Affected families are likely to spend a significant proportion of their monthly income on treatment as a direct cost of scabies [12]. However, indirect expenses have not been assessed systematically and should be a focus of future studies [12]. Disease treatment and control are particularly difficult because resource-disadvantaged populations are prone to the disease and are less likely to be able to afford effective treatment or seek appropriate medical care [7]. A study in rural Mexico suggested that scabies treatment was ineffective in over half of the cases; the total mean cost per patient was 66 new pesos (US$24) over a 6-month period [39]. Considering that the mean daily wage in the region was 15.2 new pesos (US$6) at the time, this represented a considerable financial burden. The burden was also great for pyoderma, where an average of 136 new pesos (US$52) was spent on ineffective treatments over a 6-month period [39]. Another study in rural Uttar Pradesh (India) explored the loss of income due to absence from work and cost of treatment during a peak in scabies [40]. The cost was US$5.29 per capita per year, almost 4% of the average annual income of US$113. Moreover, disease complications such as renal failure may incur extra expenses for hemodialysis or other treatments. For individuals living in remote Australian aboriginal communities, the cost was estimated as AU$100,000 per year for each person [41].

## Is global control of scabies achievable? what are the challenges?

Based on our understanding of successful previous models to control other parasitic infections, evidence suggests that scabies elimination is a realistic and achievable goal although not an easy mission. Increased awareness of the long-term health consequences of scabies and the ready availability of tools to accomplish control have brought global control of the disease within sight [11].

### Current control strategies and community-based tools

The control of scabies as well as its complications at the population level has been acknowledged as an important public health challenge in some affected countries [1]. Treating patients with scabies together with their contacts is not enough to achieve the aim of scabies elimination, and is particularly difficult due to treatment noncompliance in economically disadvantaged populations whose members are less likely to seek appropriate medical advice. Therefore, mass drug administration (MDA) is receiving more attention in light of support from recent studies that suggested that MDA campaigns hold the greatest potential benefit for these settings [42–44]. Large-scale studies to evaluate MDA with oral ivermectin compared to topical scabicidal treatment have recently received greater attention [42, 45, 46]. Experts suggest that a vital part of control programs is to incorporate them into current public health and clinical algorithms and systems [9].

### Models of successful global control programs

Lessons from past eradication programs for endemic diseases have shown that short-term control measures can be ineffective in sustainably lowering the disease burden [32, 47–49]. The ineffective use of scabicides may in turn increase the spread of drug resistance, thereby jeopardizing the success of future control efforts [32]. These factors have stimulated discussion at the highest levels for some time as to whether the global control of scabies should be attempted [32]. Control, it is believed, should be targeted at the worst-affected communities, and well-designed control studies have indicated that with sustained intervention, success is guaranteed. Frequent low-density treatment in an endemic community would yield more advantages than infrequent mass screening with treatment [50]. In addition, where the prevalence is not high enough to justify an aggressive elimination attempt but scabies is still a significant health problem, better diagnostic and treatment protocols are required to prevent futile treatment cycles that promote the emergence of resistance. Interventions should also consider the social and emotional factors of scabies that may require the participation of multi-professional experts. The complicated links between the disease and developmental issues such as housing conditions, sanitation, economic status, hygiene, nutrition, education, and maternal health are important determinants of the success of control programs. Finally, securing financial and political support is the next step toward controlling this NTD on a global scale. Reducing scabies prevalence is feasible in endemic locations only by integrating mass therapy, community involvement, health education, training for health professionals, and ongoing surveillance along with socioeconomic development in poor settings [12].

### Challenges to scabies global control

#### Challenges related to disease transmission

Because clinical signs and symptoms may take as long as 10 weeks to develop after primary infestation [51], there is a period in which an individual is asymptomatic but can transmit the disease to contacts. Persuading asymptomatic individuals to undertake unpleasant and time-consuming treatment regimens when they can perceive no personal benefit has been challenging for control programs [32, 47].

Experience has shown that programs that fail to reach the contacts of symptomatic individuals are not successful [32, 47]. Therefore rigorous community education about the necessity to treat contacts such as household members, even if not symptomatic, will be a key in designing successful control programs [47]. The importance of asymptomatic infestation should not be underestimated. This is mainly found in older, very young, and immunocompromised persons who may remain undiagnosed for a long time, and thus represent mite reservoirs [47, 49].

Environmental reservoirs must also be considered even though it is not clear to what extent *S. scabiei* can be transmitted through contaminated bedding, flooring, clothing, and furniture [32]. Because mites can survive away from their mammalian hosts for up to 36 h at 21 °C [51], transmission occurred in as many as 15% of the cases when the previous bedding occupant’s infestation comprised over 50 mites, but in only 1.33% of cases with fewer than 20 mites [52]. This implies that although environmental transmission in normal scabies infestations is probably low, it may be significant in crusted scabies. Although environmental transmission is considered minimal, and treating potential fomites is not recommended, it is important to note that topical permethrin for patients can also act as an indirect treatment of clothing and bedding. This would not be the case with the treatment with oral ivermectin on a mass level. Therefore, it is crucial to establish whether the environmental transmission of *S. scabiei* is significant enough to undermine control attempts [11]. Studies have shown that at the community level, the main risk factors for scabies infestation and heavy infection in underdeveloped regions were crowding; living together in high-density settings; recurrent body contact; sharing the same space, bed linen, and clothes; and poor housing conditions [14, 53, 54]. These findings emphasize the importance of environmental transmission of the disease.

#### Challenges related to therapy

##### Ivermectin in mass drug administration: miracle drug or double-edged sword?

Ivermectin has been successfully used to treat several hundreds of millions of individuals in various MDA programs to control parasitic diseases such as onchocerciasis and lymphatic filariasis, and is considered well tolerated with high compliance and the additional benefit of reduced scabies rates [12, 37, 42, 43].

Oral ivermectin has been considered for the epidemiological control of scabies, especially in settings where compliance with topically applied agents may pose a problem [12, 29, 37, 55]. In a Brazilian slum, scabies-related morbidity rapidly regressed after using orally administered ivermectin, with significantly decreased itching and fewer sleep disturbances after 1 week [29]. In a study from the Solomon Islands, scabies prevalence, impetigo, and hematuria (a marker of renal damage because of GAS) were remarkably reduced after ivermectin therapy [37].

Nevertheless, the utilization of oral ivermectin in MDA remains a controversial issue [12]. First of all, the drug is not registered in several countries for the indication of scabies treatment, and it is linked to some safety concerns in children younger than 5 years and pregnant women [11, 12, 42]. Second, resistance may develop after repeated, prolonged courses with oral ivermectin, as for topical compounds [56–58]. Third, some studies have shown evidence against the better effect of ivermectin over topical agents [59, 60]. However, other trials reported similar or superior therapeutic efficacy for ivermectin over various topical compounds [45, 46, 61, 62]. The results of the landmark Skin Health Intervention Fiji Trial (SHIFT) [42] of a scabies MDA program in an island community with hyperendemic scabies found significant reductions in scabies and impetigo prevalence from baseline to 1 year in all three tested groups (permethrin for patients and contacts, permethrin-MDA, oral ivermectin-MDA), but the greatest effects in the ivermectin group. Adverse events (itching or headache) were more frequent with ivermectin than permethrin (15.6% vs. 6.8%); however, all patients had mild events which resolved quickly [42].

##### Ivermectin in epidemic versus endemic situations, is it the same?

Some have proposed that orally ivermectin seems to be slightly less efficacious for treating individual patients than topical compounds such as permethrin or benzyl benzoate. They argue that ivermectin is seen as having the greatest impact in mass treatments of epidemics in closed settings such as prisons and nursing homes. This result may be due mainly to the exhaustive treatment of contacts (including all those in whom scabies mites are incubating but who remain asymptomatic) that this MDA approach facilitates. Targeting all contacts, while an essential goal, is more difficult to achieve during epidemics because of practical considerations involved in the case-by-case treatment of contacts or mass treatment with a scabicidal cream [44]. Treating only symptomatic persons along with their contacts is unlikely to result in control of scabies at the population level [8, 63].

##### Number of ivermectin doses for a positive outcome

Because unhatched mite eggs are resistant to ivermectin, it is a good decision to prescribe another dose 1 or 2 weeks later, not only for symptomatic patients but as MDA to prevent the emergence of resistance [64]. The significance of this second dose for healthy individuals still awaits evaluation, as do the diagnostic and epidemiologic schemes that determine when to initiate an MDA program, the time interval between interventions, and when to stop the interventions [64].

##### MDA and drug resistance

To avoid resistance to currently effective drugs, caution should be exercised with the mass use of scabicides [32]. In vitro resistance to topical compounds has been demonstrated, and clinical resistance to ivermectin has been shown since 2004 [56, 58]. Although cases of reported resistance are infrequent, they can occur. Providing two directly observed doses 7 to 14 days apart in a large-scale MDA, regardless of whether clinical scabies is present, may decrease the progress of possible ivermectin resistance [42].

##### MDA and drug toxicity

The safety of ivermectin was notable in previous MDA control programs [42]. However, this drug is contraindicated for patients with *Loa loa* filariasis, and its use in pregnant women and small children is questionable [42]. Mild adverse events (itching or headache) were more common in patients on ivermectin than permethrin, but resolved quickly [42]. Permethrin is favored in children with a body weight less than 15 kg, in pregnant and breast-feeding women, in patients on warfarin, and in patients with neurological diseases and on anticonvulsant drugs [42]. The controversy regarding ivermectin safety thus requires further investigation.

##### MDA success and improved living condition

One may claim that scabies control is a long-term goal requiring not only MDA programs, but community empowerment, attention to environmental determinants, and guaranteed access to effective primary health care. Implementing MDA programs to control NTD is not an alternative to improvements in living conditions and ensuring safe water, sanitation, and better education for the entire population, which would consequently be reflected as better control of NTDs including scabies [65]. Relying only on MDA, especially if not implemented appropriately, may fail to break disease transmission and produce the large, optimistic outcomes anticipated for the global control of NTDs at least within the target date [65].

#### Challenges related to diagnostic tools

Diagnostic methods for scabies have important limitations. To date, the diagnosis relies on identification of clinical signs and microscopic examination of skin scrapings. However, typical skin lesions and burrows are not easy to observe and are usually masked by eczematous changes and impetigo. These features can confuse the picture with that of several other diseases and conditions such as bacterial impetigo and folliculitis, tinea, pityriasis rosea, viral exanthem, insect bites, eczema, contact dermatitis, papular urticaria, psoriasis, onchodermatitis, and bullous pemphigoid [66]. In addition, microscopic inspection of skin scrapings has a low sensitivity of only 50%, due in part to the low density of mites in ordinary scabies [66]. Indian ink staining was described as impractical, and thus is rarely used. Dermatoscopy is also used for diagnosis, but its performance, feasibility, and expense have limited its usage [13, 67]. Therapeutic tests have been tried; however, misinterpretation of positive and negative responses after treatment and their time-consuming nature are disadvantages of these methods [13]. Like microscopy, PCR has low sensitivity due to the small mite numbers. As a complement to PCR testing, ELISA has been added to evaluate results, but this needs extra time and effort [68]. To date, the difficulty in obtaining a culture system to collect adequate quantities of *S. scabiei* organisms has hindered the development of intradermal tests and other immunological assays. Animal models have tried to obtain whole-mite extracts, but the products were crude, inconsistent in potency and composition, and contained a combination of host antigens together with other cross-reactants of house dust mites [66]. Thus far, ELISA has been developed only for animal scabies. The antigens in this kit include whole-mite extract from pigs (*S. scabiei* var. *suis*) and red fox itch mites (*S. scabiei* var. *vulpes*). However, the sensitivity of this assay is 48% in humans vs. 80% in pigs and 84% in dogs, with low specificity due to cross-reaction [68]. Rapid point-of-care tests for scabies diagnosis are not available at present [69]. Immunoassays either in the form of ELISA or rapid diagnostic tests for antibody detection in humans will need to be developed as a means of mass screening in high-risk populations. These assays would provide information about the extent of disease exposure and the impact of starting MDA cycles in affected communities, and would make it possible to monitor the effects of MDA on a regular basis, e.g., once a year.

Due to diagnostic limitations especially in poor settings, where variable disease presentations are common and experience with diagnostic tools maybe lacking, it has been suggested that a simple, reliable diagnostic algorithm may constitute a helpful diagnostic approach [11]. Mahe and colleagues have developed a highly accurate clinical diagnostic algorithm usable by non-experts, based on patient history and examination for common skin diseases in sub-Saharan Africa [70]. Good news was also announced in 2020 regarding the Consensus Criteria for the Diagnosis of Scabies [71]. In 2020, the IACS published the criteria that underpin a pragmatic and rigorous diagnostic scheme of clinical features and methods which, it is hoped, will guide the standardization of scabies diagnosis [71]. The criteria were established in a Delphi study involving global experts and classify the diagnosis of scabies in three levels of certainty (e.g., confirmed, clinical, or suspected scabies) with eight subcategories. Level A (confirmed scabies) relies on the direct identification of *Sarcoptes* mites or their products. Level B (clinical scabies) and level C (suspected scabies) are based on clinical signs and symptoms. The criteria are intended to be applied in a range of settings, including research, clinical situations, and public health, by using the appropriate level and subcategories of diagnosis [71]. Although the criteria represent a good opportunity for a standardized diagnosis of scabies, the developers continue to plan validation studies, and foresee future efforts to develop training materials and implement survey methods [71].

##### Diagnostic research challenges and updates

Improving diagnostic tools for humans mainly requires sufficient number of mites to prepare antigens suitable for immunodiagnostic assays. The low numbers of parasites in infected patients and the absence of an in vitro culture method for mites have been the obstacles to diagnostic research. Research on recombinant allergens of *S. scabiei* has been under way since 1997 and is ongoing, with promising results [72, 73]. Many molecules similar to house dust mite allergens and other homologous DNA sequences have been identified [74–77], and it is hoped that recombinant scabies allergens will hold potential to improve scabies diagnosis and immunotherapy [66].

#### Challenges related to vaccine development

Generating a vaccine to scabies is seen as a real possibility for many reasons [6]. The mite induces both innate and long-term adaptive immunity in infected patients [75]. Immunity develops rapidly, with higher levels of host antibodies and reduced mite numbers on subsequent infection [78, 79].

Research has shown that vaccination against scabies may induce various degrees of immune response and protection in animals, including rabbits [80], goats [81], sheep [82], and mice [83]. Various antigens have been tried alone or in combination, with promising results [79–85]. Whole-body extracts from *S. scabiei* var. *canis* [80], *Psoroptes* sp. scabies mites combined with the related species *P. cuniculi* (rabbit scabies), and *P. ovis* (sheep scabies) have shown different degrees of immune response and protection [82, 85]. In addition, the recombinant antigens Ssag1 and Ssag2 from *S. scabiei* var. *hominis* [84], a DNA vaccine for *S. scabiei* var. *cuniculi* which encodes for paramyosin [83], and extract mixtures of equal amounts of different house dust mite species (*Dermatophagoides farinae*, *D. pteronyssinus*) [79] have been tried.

The data available thus far show that vaccine development against scabies seems a realistic objective to protect both humans and animals. However, factors such as the type of antigen or antigen mixture used require further investigation. Factors that can determine the success or failure of these efforts include the immunization schedule, the amount or dose of antigen challenge, complementary treatment with adjuvants, and the route of body entry. Recombinant technology holds promise to produce a vaccine with one or more key antigens or an antigen cocktail. Nevertheless, all determinants of vaccination protocols need further research to formulate the most effective methods [6].

## What do we need to do for effective scabies control? Future research plan and other needs

Many important gaps remain in research on scabies [86]. In terms of treatment, areas of concern include firstly the limitations of available therapeutic options and safety. There are concerns regarding ivermectin and other drugs in small children and during pregnancy, and dose optimization for ivermectin is an area that awaits further study. Secondly, further work is needed on the appearance of mite generations resistant to different scabicidal drugs, the best treatment for crusted scabies, and the low effectiveness of available treatments in preventing relapses. A final area awaiting progress is vaccine development [6, 87]. Furthermore, guidelines should be established for the best therapeutic options to manage complications such as inflammatory skin responses, secondary bacterial infections, and others. Comparative studies to assess new drugs should be encouraged. Although still in the preclinical phase in animals, research on single-dose moxidectin by Bernigaud and colleagues is an example of such efforts [88]. Further work is needed to assess the effectiveness of MDA as a control tool for scabies on a community-wide scale. The recent Fiji islands SHIFT study [42] is a role model for assessing ivermectin and permethrin in MDA programs; evaluations beyond island settings and in larger populations are needed. Key issues include the frequency of cycles needed, the delivery methods, evaluations of effectiveness, feasibility and patient satisfaction, cost-effectiveness, and the impact of disease and impetigo control on other serious complications [89].

To identify unique social determinants of their effectiveness, research is needed on population-based interventions in different settings. For example, involving traditional practitioners in efforts to eliminate scabies (with a focus on the outcomes and challenges such approaches may face) is an important area for future research [90]. In addition, the effectiveness of long-lasting insecticide-impregnated nets and the cost-effectiveness of MDA and other screening and intervention programs are other areas for further work. Disease mapping in neglected areas (for example, in poor communities in Africa) is also needed [11].

The economic impact of scabies needs further consideration to determine both its direct and indirect costs [12]. In addition, the long-term effects of childhood scabies on the development of rheumatic heart diseases and other infections warrant additional investigation [11]. In addition, funding organizations need to reallocate their resources toward scabies, in the realization that scabies is a disease of the poor. Priority should be given to funding research on diagnosis, treatment, and various population-based tools.

In 2020, and in light of the needs described above, scabies was included in the updated WHO roadmap for neglected tropical diseases 2021–2030, a promising step in the progress toward scabies control [2]. In this roadmap, scabies is targeted for control rather than elimination. Although the terms “control and elimination” are sometimes used changeably, to reflect the same meaning of ending the endemicity of a disease or a pathogen in a setting, the roadmap has settled specific definition to each of the terms. Disease control is defined as the “reduction of disease incidence, prevalence, morbidity, and/or mortality to a locally acceptable level as a result of deliberate efforts,” and the roadmap notes that “continued intervention measures are required to maintain the reduction” [2]. Elimination on the other hand is defined as the “reduction to zero of the incidence of the incidence of infection by a pathogen in a defined geographical area with minimal risk of reintroduction, as a result of deliberate efforts,” and the roadmap notes that “continued action to prevent re-establishment of transmission may be required” [2]. One indicator of progress toward the scabies control target of the roadmap will be expressed as the number of countries that have involved scabies management in their universal health coverage package. The baseline number in 2020 is zero, and it is hoped that this indicator will increase to 25 (13%) in 2023, to 50 (26%) in 2025, and to 194 (100%) countries in 2030. Impact indicators are defined as the number of countries that have used MDA in all endemic regions, starting from zero at baseline in 2020 and increasing to 3 in 2023, to 6 in 2025, and to 25 countries in 2030. The roadmap specifies important critical actions needed to attain the required targets [2]. These include (1) developing tools and guidance for mapping to assess the burden of scabies in endemic countries, (2) developing guidance for implementing preventive chemotherapeutic campaigns (with MDA), and (3) developing a funding plan and support system, securing financial resources for ivermectin and other topical therapeutics, and advocating for incorporation in universal health coverage.

The WHO has ensured that the map and its indicators will be continuously monitored, revised, and evolved to update formats based on progress made on the ground. According to the WHO, the roadmap is hoped to be a call for action for different countries, decision makers and policy makers, funders, researchers, and disease experts to collaborate and interconnect their plans and strategies in order to create aligned efforts toward the control and eradication of NTDs, and thereby reduce the suffering of poor populations affected by them. The goal of the integrated approach in this roadmap to scabies as an important dermatological NTD is to reduce morbidity, disability, and the psychosocial impacts of this and other debilitating diseases [2].

Good news and progress can be anticipated because of the recent recognition of scabies as an NTD by the WHO, and its incorporation into the roadmap for neglected tropical diseases 2021–2030. It is anticipated that highlighting scabies in this way will attract the attention of all who are concerned about the vital need to advance research and align efforts aimed at eliminating this disease.

## Conclusions

Scabies, an important parasitic infectious skin disease, was recently recognized by the WHO as an NTD and included as part of the WHO roadmap for NTD 2021–2030. Nevertheless, efforts to eliminate this disease still require us to perceive it more widely as a population-level health challenge that merits attention in many settings worldwide. Further research on scabies is recommended with a view to improving control efforts. Additional research priorities are to understand and overcome existing problems that may hamper global control efforts such as insensitive diagnostic tests and controversies regarding therapeutic options and vaccination challenges. The extremely effective treatment with orally administered ivermectin may open a promising new chapter in scabies control through MDA campaigns, along with mapping the disease prevalence to ensure successful large-scale programs.

Despite the current challenges, scabies global control is now within reach. With sustained interventions, continuous resources, sincere commitment and support, and a focus on the targets and indicators identified in the WHO roadmap for NTD 2021–2030, scabies global control appears to be a worthwhile, realistic goal that is potentially achievable in the not so distant future.

## Search strategy

References for this nonsystematic narrative literature overview and commentary were collected by searching PubMed, Scopus, and Web of Knowledge (mainly PubMed) up to December 2017 for major articles and their reference sections. The search was subsequently extended to locate relevant items published up to 15 November 2020. The objectives were to explore major developments in scabies, along with recent practices and updates in its epidemiology. In addition, the literature search aimed to retrieve information on control efforts, challenges and experiences, on knowledge gaps, and on needs assessments and recommendations for the future in the field of global scabies control. The search terms used were “scabies”, “*Sarcoptes scabiei*”, “elimination”, “control programs”, “neglected tropical diseases”, “epidemiology”, “population-based intervention”, “challenges”, “research updates”, “diagnosis”, “treatment”, “mass drug administration”, “ivermectin”, “vaccine”, and combinations of these. The initial search and screening of all items were carried out by the contributor (OME), and then the author (AAE) re-assessed the content of all papers. Final inclusion was subjectively restricted to 90 major general medical and epidemiological articles based on their relevance to the study objectives and aims. Preference was given to review articles, large epidemiological field studies, and articles that comprehensively and/or appropriately covered the topics of interest and helped explain the significance of the issues this article covers. Additional articles were identified by visiting relevant websites such as those of the WHO, the International Alliance for the Control of Scabies (IACS), those concerned with NTDs, and major journals. No language restrictions were used.

## Data Availability

Not applicable

## Abbreviations

WHO: World Health Organization
NTD: Neglected tropical diseases
IACS: International Alliance for the Control of Scabies
STAG: Strategic and Technical Advisory Group
MDA: Mass drug administration
SDGs: Sustainable Development Goals
YLDS: Years lived with disability
DALYs: Disability-adjusted life-years
GBD: Global Burden of Disease
GASP: Group A *Streptococcus*
SHIFT: Skin Health Intervention FIJI Trial
PSGN: Post-streptococcal glomerulonephritis
